# Immunoregulatory framework and the role of miRNA in the pathogenesis of NSCLC – A systematic review

**DOI:** 10.3389/fonc.2022.1089320

**Published:** 2022-12-21

**Authors:** Nikhil Samarth, Pooja Gulhane, Shailza Singh

**Affiliations:** National Centre for Cell Science, NCCS Complex, Ganeshkhind, SP Pune University Campus, Pune, India

**Keywords:** NSCLC, EGFR, miRNA, autophagy, synthetic biology, EGFR-TKIs

## Abstract

With a 5-year survival rate of only 15%, non-small cell lung cancer (NSCLC), the most common kind of lung carcinoma and the cause of millions of deaths annually, has drawn attention. Numerous variables, such as disrupted signaling caused by somatic mutations in the EGFR-mediated RAS/RAF/MAPK, PI3K/AKT, JAK/STAT signaling cascade, supports tumour survival in one way or another. Here, the tumour microenvironment significantly contributes to the development of cancer by thwarting the immune response. MicroRNAs (miRNAs) are critical regulators of gene expression that can function as oncogenes or oncosuppressors. They have a major influence on the occurrence and prognosis of NSCLC. Though, a myriad number of therapies are available and many are being clinically tested, still the drug resistance, its adverse effect and toxicity leading towards fatality cannot be ruled out. In this review, we tried to ascertain the missing links in between perturbed EGFR signaling, miRNAs favouring tumorigenesis and the autophagy mechanism. While connecting all the aforementioned points multiple associations were set, which can be targeted in order to combat NSCLC. Here, we tried illuminating designing synthetically engineered circuits with the toggle switches that might lay a prototype for better therapeutic paradigm.

## 1 Background

**Figure 1:**
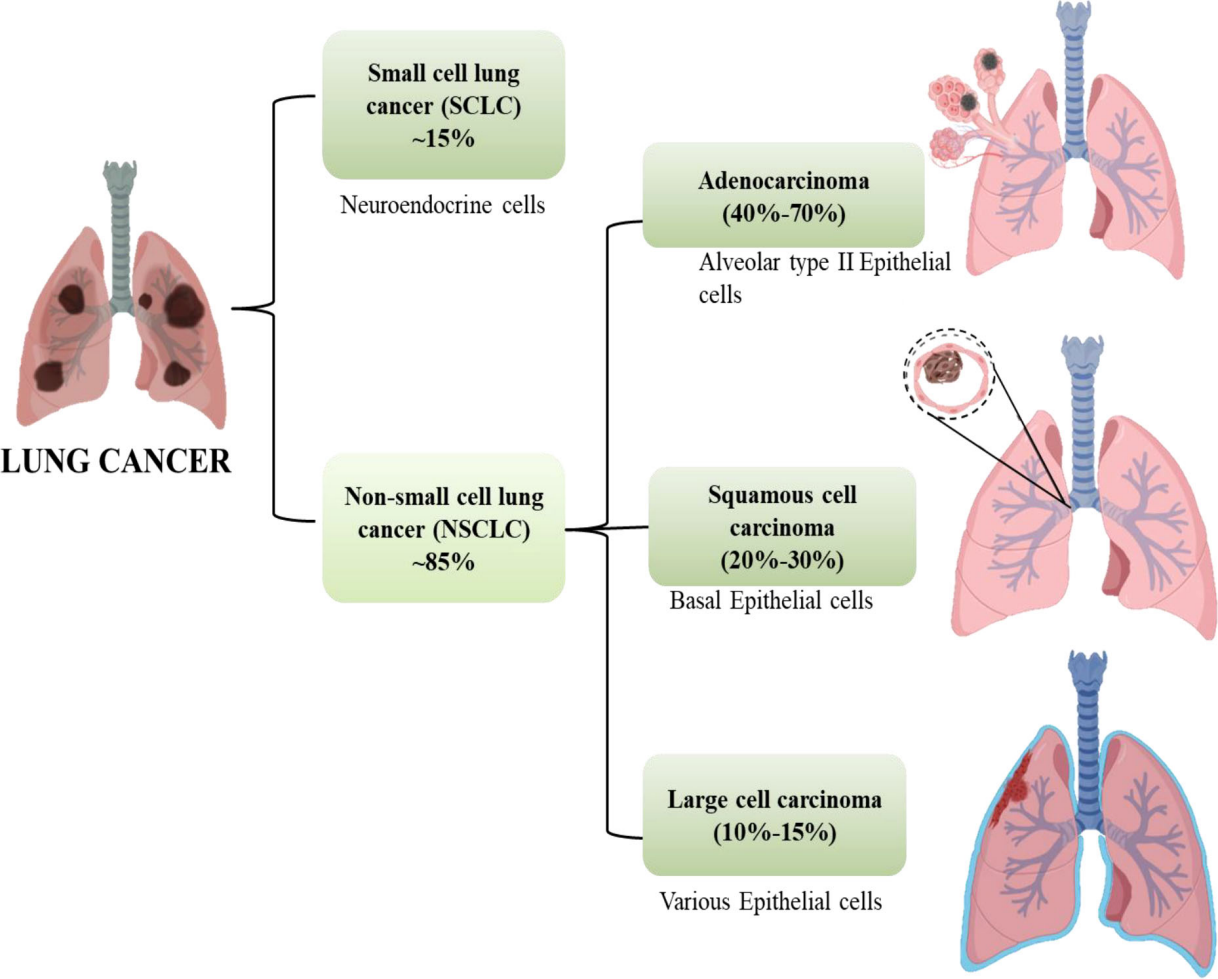
Subtypes of lung cancer.

Cancer is anticipated to rank as the top cause of death and the single biggest obstacle to raising life expectancy in every country over the globe in the 21^st^ century. Non-communicable diseases (NCDs) nowadays account for the majority of fatalities globally. According to World Health Organization (WHO) estimates from 2019, cancer is the first or second leading cause of deaths in 112 out of 183 countries (1). Worldwide, the incidence and death of cancer are rising quickly. Cancer transitions are especially noticeable in rising nations, where the disease is getting worse and the organization of frequent cancer types is changing at the same time. These cancers are frequently attributed to a so-called modernization of lifestyle, but the various cancer profiles in different nations and regions show that there is still significant geographic diversity, with local risk factors persisting in populations going through very different stages of social and economic development. A revision to the GLOBOCAN 2020 projections of cancer incidence and mortality provided by the International Agency for Research on Cancer, the number of new cancer cases worldwide is envisaged to be 19.3 million, and the number of cancer fatalities occurred to be close to 10.0 million. Lung cancer is the second most prevalent cancer diagnosed and the main cause of cancer mortality in 2020, accounting for roughly one in ten (11.4%) cancer diagnoses and one in five (18.0%) fatalities, with a predicted 2.2 million new cancer cases and 1.8 million deaths (1, 2). For both men and women, lung cancer is one of the most hazardous cancers. The fatality rate is higher than the sum of colon, breast, and pancreatic cancers. More than half of patients with lung cancer die within a year of being diagnosed. Lung cancer’s poor prognosis is a result of late diagnosis because there aren’t any obvious signs during the early stages of the disease’s progression. Lung cancers are divided into two subgroups according to their histological characteristics: non-small-cell lung cancer (NSCLC, which accounts for 85% of cases) and small-cell lung cancer (SCLC, which accounts for 15% of cases). Squamous-cell carcinoma, adenocarcinoma, and large-cell carcinoma are the three primary histological subtypes of NSCLC. Adenocarcinoma accounts for 40% to 70% and instances in the outer portion of the lung. Squamous-cell carcinoma accounts for 20%–30% of lung cancer cases and is typically seen in the centre of the lung and the remaining 10% -15% cases are of Large cell Carcinoma which can be found throughout the lung as shown in **Figure 1** (3).

## 2 Molecular events leading to pathogenesis in NSCLC

**Figure 2:**
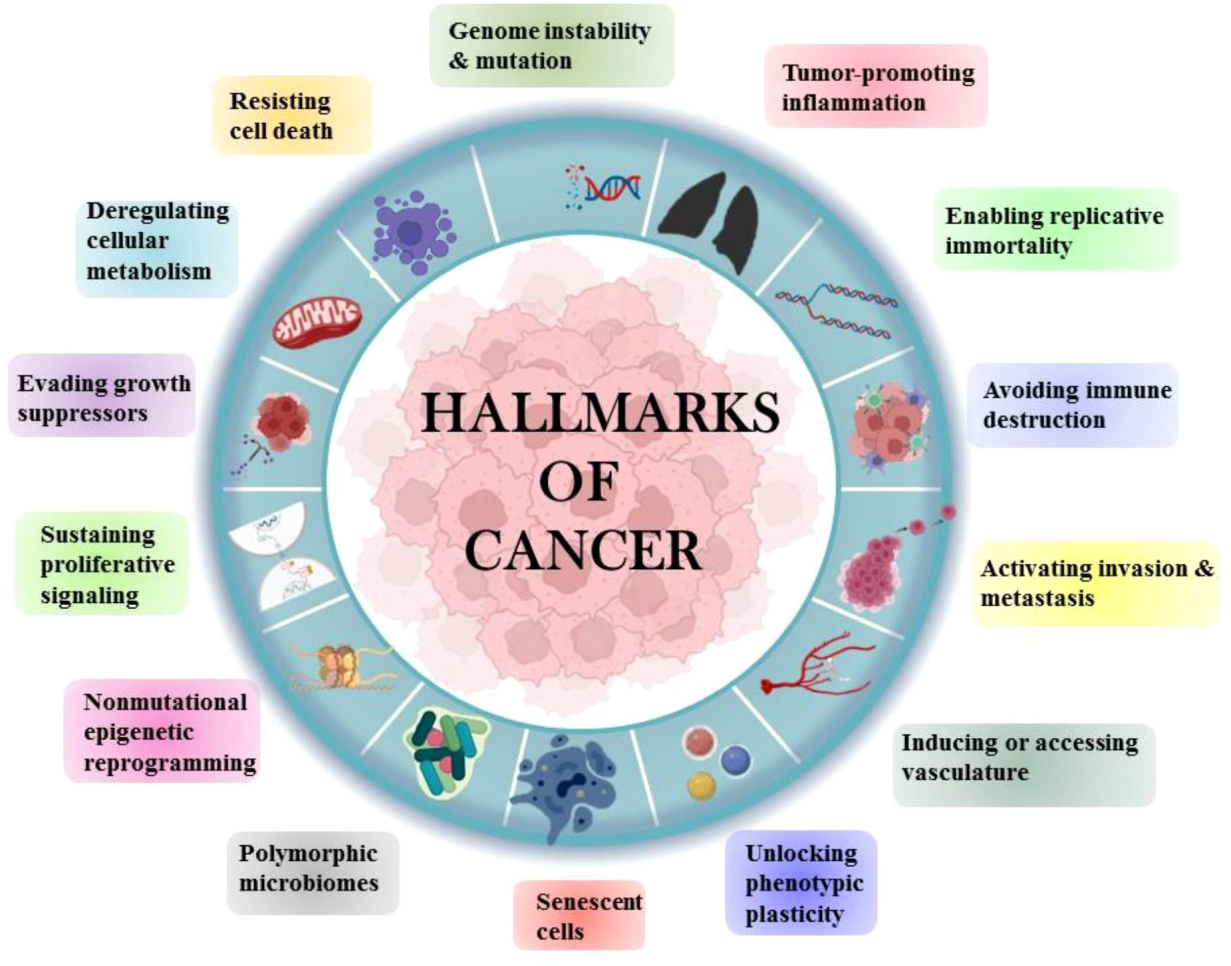
Hallmarks of Cancer.

Tobacco has been a part of the cultural and economic structure since antiquity. Cigarette smoking was thought to be more important than occupational exposure in the general population in the cause of NSCLC. Cigarette smoking had a far greater impact than any other risk factor for lung cancer (4). The majority of NSCLCs are diagnosed in ex-smokers, implying that the accumulation of molecular damage caused by cigarette smoke sets in motion a chain of events that leads to cancer development even decades after smoking cessation (5). Human cells acquire a set of functional abilities as they transition from normal development states to neoplastic growth states. More specifically, these abilities are essential for human cells’ capacity to produce malignant tumors. The genetic and molecular changes that occur during carcinogenesis result in the “hallmarks of lung cancer” (**Figure 2**).

### 2.1 Sustaining proliferative signaling

Numerous growth factors and their receptors are overexpressed by NSCLC cells and surrounding stromal cells, resulting in autocrine and paracrine growth stimulation loops. During tumor development proto-oncogenes encode several growth factors and receptors which are activated by various mechanisms. RAS, a member of an important signal transduction pathway, is one such proto-oncogene. The KRAS gene is mutated in 15% to 20% of all NSCLCs and approximately 30% of lung adenocarcinomas, with the most common mutation occurring at codon 12 (5). In NSCLC, two tyrosine kinase growth factor receptors, EGFR and Her2/Neu, are frequently overexpressed. EGFR and HER2/NEU somatic mutations have recently been reported in patients with LUAD (lung adenocarcinoma) among females and non-smokers (6, 7). Shigematsu et al. found that all mutations occurred within exons 18-21 of the EGFR tyrosine kinase domain (8).

### 2.2 Evading growth suppressors

For cancer cells to keep growing, it is imperative that they avoid antigrowth signals and must inhibit tumour suppressor genes (TSG), which are responsible for antigrowth signals. Cancer cells were found to have mutations in tumour suppressor genes, with p53 being the most frequently mutated one (9). More than 50% of patients with NSCLC have mutations that render the p53 tumour suppressor gene inactive (10). In addition, TSGs like RB1, CREBBP, KEAP1, STK11, CDKN2A, NOTCH1, and PTEN are particularly relevant to NSCLC. These TSG mutations typically exclude one another, showing that different genes can contribute to the development of cancer (11).

### 2.3 Avoiding immune destruction

The immune system’s capacity to restrain itself from attacking healthy body cells is a crucial component. This is accomplished by “checkpoint” proteins on immune cells, which function as switches that must be turned on (or off) in order to initiate an immune response. These checkpoints are sometimes used by cancer cells as a defence mechanism against immune system attacks. A group of receptors on the surface of activated T cells make up immune checkpoints. Immune checkpoint molecules prevent self-tolerance and immune homeostasis under “normal” physiological circumstances where it is important to prevent collateral tissue damage. However, by applying the “brakes” to host immune activation, they help to tolerate tumour cells (12, 13). More than 20 immune checkpoint modulators have been described for this mechanism, but programmed cell death receptor 1 (PD-1) and cytotoxic T lymphocyte associated protein-4 (CTLA-4) are particularly well-versed (14, 15). PD-L1, an immunosuppressive protein, is overexpressed in lung tumors, according to accumulating evidence, and inhibiting this pathway has provided long-lasting relief for some patients with advanced-stage NSCLC (16).

### 2.4 Enabling replicative immortality

A defining trait of tumour cells is replicative immortality or telomerase activation (17). The level of telomerase activity has been found noticeably higher in lung tumour cell lines in all histopathological subtypes of the tumor (18). According to the data, more than 80% of the NSCLCs studied expressed telomerase activity. Telomerase can stimulate tumour progression by ensuring the maintenance of telomeres above a critical short length, thereby preventing the induction of apoptosis or cellular senescence (19, 20). Chromosome instability brought on by telomere dysfunction may activate telomerase, activate oncogenes, and/or silence tumour suppressor genes, all of which work together to promote cancerous transformation, tumour growth, and drug resistance (21, 22).

### 2.5 Tumor promoting inflammation

When healthy cells are killed off by tumour cells, it results in inflammation that promotes the growth of the tumor. This causes the pro-inflammatory signals to be released from the beneficial cell contents into the interstitial tissue. This state will last as long as tumour cells grow (23). According to their cause, mechanism, severity, and outcome, the various types of inflammation contribute to the development of cancer, such as inflammation due to infection, environmental exposure, therapy induced inflammation and tumor associated inflammation. Tobacco smoke, silica-containing products, and asbestos can all increase the risk of developing NSCLC because the immune system is unable to get rid of these three substances, chronic inflammation will continue and as the inflammation progresses, tumors will form, migrate, grow, and differentiate from normal cells into different types of tumour cells (24).

### 2.6 Activating invasion and metastasis

When tumour cells develop the capacity to infiltrate the surrounding tissues, the invasion process is triggered as these motile cells pass through the extracellular matrix and basement membrane, developing into intravasation as they infiltrate the lymphatic or vascular circulation. The development of secondary tumors in distant organs results from a series of steps in the tumour metastasis process. These metastatic cells then travel through the circulatory system, invading the extracellular matrix and vascular basement membrane as part of the extravasation process, which is largely to blame for the mortality and morbidity associated with cancer (25). In NSCLC, the brain is one of the primary sites of lung carcinoma metastasis. Adenocarcinomas with epidermal growth factor receptor (EGFR) mutations and EML4ALK1 rearrangement are more frequently found to have brain metastases (26, 27). Metastatic NSCLC cells had elevated levels of the cell adhesion protein CD15 as TGF-α activates E-selectin (CD62E), it is also discovered to be overexpressed in hCMEC/D3 human brain endothelial cells. This raises the likelihood that metastatic NSCLC cells will attach to the brain endothelium, which will allow tumour cells to invade the tissue (28).

### 2.7 Inducing/accessing vasculature

The term “angiogenesis” refers to the process by which new blood and lymphatic vessels develop from an already-existing vasculature. This gives tumour cells the ability to take in nutrition in the form of nutrients and oxygen as well as the ability to expel metabolic waste. Solid tumors require a blood supply in order to develop beyond a few millimetres in size, and that is why angiogenesis is essential to the process of tumorigenesis (29). In relation to tumour vascular density, cancer metastasis, and prognosis, VEGF is the most potent and selective growth factor for endothelial cells. Non-small cell lung cancer patients have been shown to have high levels of circulating VEGF (30, 31). It is a growth factor that encourages vascular endothelial cells to proliferate and migrate while also inhibiting apoptosis and regulating their permeability. In NSCLC, it contributes to tumour growth by lymphangiogenesis, neoangiogenesis, lymph node spread, and high levels of VEGF have been linked to a poor prognosis (32, 33).

### 2.8 Genome instability and mutation

Several genes governing cell division and tumour suppression are damaged as a result of an increased propensity for genomic alterations and mutations brought on by the abnormal proliferation of cancer cells termed as Genomic instability. Since survival-enhancing mutations enhance the likelihood that those mutations will spread to subsequent cells, genomic instability has a tendency to accumulate in cancer cells (34). According to genomic research, Lung cancer has thousands of somatic mutations, copy number changes, and genome duplications. Due to exposure to tobacco carcinogens and germline genomic instability, lung cancer has a high somatic mutation rate, also the NSCLC initial tumors have a significant level of genetic variability (35, 36). Oncogenes, tumour suppressors, cyclins, cyclin-dependent kinases, and defects in cell cycle regulation are the main molecular mechanisms generating genomic instability in lung cancer. Genomic instability is also a result of defective spindle assembly checkpoint components including Bud and Mad family members, as well as aberrant DNA repair processes like BER, NER, TLS, HR, NHEJ, and MMR (37).

### 2.9 Resisting cell death

The systems used by cancer cells to detect damage or abnormalities may be changed, which would stop the appropriate signaling and activation of apoptosis. Cancer cells may potentially introduce flaws in downstream signaling or apoptosis-related proteins, which would also interfere with the normal apoptosis. Normally, the build-up of genetic abnormalities necessary to promote unchecked cell cycle progression and cancer would cause a cell to go through apoptosis. A crucial characteristic of cancer is its capacity to prevent cells from dying during apoptosis. Cancer cells harbour apoptotic defects, which are the root cause of treatment resistance in addition to carcinogenesis. It is possible that a deficiency in apoptotic signaling is the cause of NSCLC cells’ resistance to a variety of cytotoxic treatments (38). The “death domain,” a distinctive cytoplasmic region shared by members of the TNF receptor family, is essential for transferring the death signal from the cell’s surface to intracellular signaling pathways. Multiple degrees of death receptor signaling dysfunction can be present in human malignancies. These receptors’ surface expression can be down regulated or even completely missing at the receptor level, which prevents the death signal from reaching intracellular signaling cascades from the cell surface. Numerous tumours have been shown to have an increase in the ratio of anti- to pro-apoptotic Bcl-2 proteins, which has been linked to tumour cell survival and apoptosis resistance (39).

### 2.10 Deregulating cellular metabolism

Cancer cells have altered cellular metabolism because they require a large amount of energy to grow rapidly. Aerobic glycolysis has an aberrant role in the energy production of cancer cells. They also have other abnormal metabolic traits, such as enhanced fatty acid production and elevated rates of glutamine metabolism, in addition to their dependence on glycolysis. Recent research has connected a number of characteristics of cancer cells, including dysregulated Warburg-like glucose metabolism, fatty acid production, and glutaminolysis, to therapeutic resistance in the treatment of cancer (40). In growing cells, glucose is a vital nutrient needed for numerous metabolic pathways. It’s possible that cancer gains from a lot of glucose. In true scenario, greater incidence and fatality rates in several forms of cancer, including NSCLC, have been connected to high blood sugar levels and a clinical diagnosis of diabetes (41). Given that the cancer cells can derive vital metabolites and energy primarily from glucose fermentation, even under aerobic conditions, Warburg hypothesized that hyperglycemia might hasten the development of cancer cells (42). Tyrosine kinase oncogenic activity has been found to be the primary driver of certain advanced NSCLC cancers. Growing evidence suggests that these kinases also cause metabolic alterations in tumour cells. Numerous studies have focused on the precise mechanisms by which oncogenic EGFR signaling rewires the metabolism of NSCLCs, and it is known that these pathways affect the control of glucose metabolism (43).

### 2.11 Emerging hallmarks and enabling characteristics

#### 2.11.1 Unlocking phenotypic plasticity

Cells ability to differentiate is typically limited in comparison to the massive amount of development and differentiation that occurs during organogenesis. This constraint allows cells to remain organized and functional within their tissue. Cells in cancer, on the other hand, go through molecular and phenotypic changes that allow them to take on different identities along a phenotypic spectrum known as cellular plasticity. Changes in cellular phenotype are important in cancer progression because they can aid in tumor initiation, metastasis, immune invasion, chemoresistance, and tumour progression (44, 45). There are three types of phenotypic plasticity that can be activated during cancer. Dedifferentiation occurs when a normal cell reverts to a progenitor-like state. Blocked differentiation occurs when differentiated progenitor cells are prevented from entering a non-proliferative state. Trans differentiation occurs when cells that were previously committed to one differentiation pathway switch to another (46, 47).

#### 2.11.2 Non mutational epigenetic reprogramming

Permanent genetic alterations, such as point mutations, deletions, translocations, amplifications, and epigenetic modifications that impact various chromatin-dependent processes, including histone modifications, DNA methylation patterns, and microRNA regulation, are the cause of the onset and progression of lung cancer. Over 50% of all human gene promoter sequences contain CpG dinucleotide islands, which make up just 1% of the human genome yet are sites of DNA methylation in eukaryotes. Increased mitotic recombination and consequent chromosomal instability are brought on by a substantial decrease in cytosine methylation at these repeated regions in cancer cells. In addition, abnormal cytosine methylation affects the transcriptional regulation of other genes involved in processes including DNA repair, apoptosis, the epithelial-mesenchymal transition (EMT), cellular motility, invasion, and metastasis (48, 49).

#### 2.11.3 Polymorphic microbiomes

The commensal microbiota has come to light as an important biomarker and regulator of tumorigenesis and cancer therapy response. Tumor-associated dysregulation of the local microbiome in the lung has been discovered in lung cancer patients and mouse models, which influences cancer progression by shaping the tumor microenvironment and modulating the activity of tumor-infiltrating immune cells. Lung microbiota has the ability to regulate specific oncogenic pathways that are directly responsible for carcinogenesis. It is possible that changes in bacteria-derived molecules caused by dysbiosis in the tumor microenvironment can affect lung cancer cell metabolism and oncogenic signaling (50, 51). The expression of genes involved in the PI3K and ERK1/2 signaling pathway was shown to increase after the human lung adenocarcinoma cell line A549 was stimulated *in vitro* with bacterial products isolated from lung cancer patient enriched bacteria. This result was consistent with the transcriptomic changes seen in lung cancer patients compared to healthy people (52). Upregulation of the PI3K pathway has been identified as an early event in the process of lung tumorigenesis, implying a direct link between lung microbiota and oncogenesis (53).

#### 2.11.4 Senescent cells

Cellular senescence is a long-lasting and irreversible cell cycle arrest with secretory features, macromolecular damage; altered metabolism that occurs in response to various stresses and more often activates a persistent DNA-damage response. It is usually viewed as an endogenous tumour suppressor mechanism due to the induction of a long-lasting and generally irreversible cell cycle arrest. Senescent malignant and non-malignant cells, on the other hand, remain metabolically active and can secrete a slew of mainly pro-inflammatory cytokines, chemokines, growth factors, and matrix-remodeling proteases known as the senescence-associated secretory phenotype (SASP). This SASP shields malignant cells from immune clearance and influences the tumour microenvironment and ultimately promotes cancer relapse and metastasis (54–57).

## 3 Tumor microenvironment

**Figure 3:**
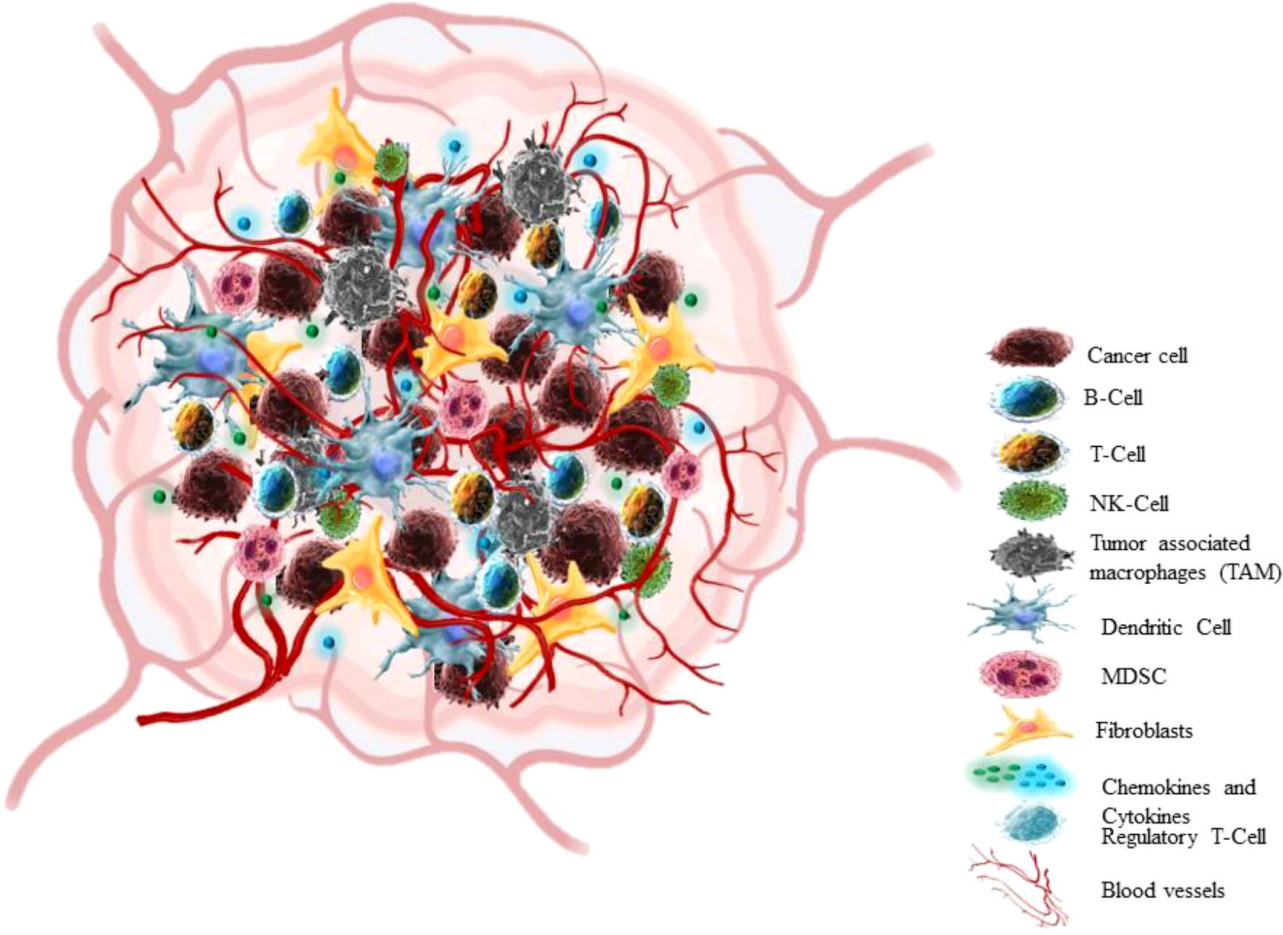
Tumor microenvironment.

In the past 25 years, a breakthrough change has occurred in the approach to cognizance biology of solid tumors. Earlier the focus of cancer research was almost restricted to some aspects like individual tumor cells, the transformation processes and mechanisms that conveyed their malignancy, but recent research has centred towards tumour in its whole complexity (58) Cancers are not just masses of malignant cells but sophisticated reprobate organs, which attract the other cells and alter them (59). TME consists of cellular (cancer cell, stromal fibroblast, endothelial cell, immune cells) and non-cellular components (ECM components) (**Figure 3**) (60). The interaction between Tumour cells and microenvironment (TME) plays a crucial role in cancer progression (NSCLC) (61, 62). The cancer cell communicates with the microenvironment through ECM, also cell-cell contacts or *via* mediators which allow these contacts such as cytokines, enzymes, chemokines, and exosomes (63).

A tumor microenvironment has four characteristics such poor nutrients, hypoxia, high acidity and the immunosuppressive microenvironment (64). Tumor cells also show deregulated metabolism due to both intrinsic (such as the genetic makeup of cancer cells) and extrinsic factors (such as oxygen tension, nutrition availability, and pH). The cancer cells scavenge nutrients in the microenvironment leading to suppression of antitumor immune response (65). Thus, the reprogrammed metabolic pathway in cancer cells satisfies particular requirements for energy, biomass, redox homeostasis, and cellular communication (66). The NSCLC is a heterogeneous tumor at genetic and cellular level (62). Numerous studies have characterized TME of lung tumors at a single cell resolution. Lambrechts et al., reported 52,698 cells in TME transcriptome using scRNA seq, out of these 75% cells originated from the lung tumor and also presented thorough classification of stromal cells with various pathways in NSCLC patients (67). Fengying Wu et al, also analysed tissue biopsy sample of 42 NSCLC patients particularly from stage III/IV using scRNA seq, in order to study tumor heterogeneity in advanced NSCLC (68). These studies have enlisted different immune cells in the NSCLC microenvironment.

### 3.1. Cancer-associated fibroblasts

Cancer-associated fibroblasts (CAFs) are a very diverse population of stromal cells that play a significant role in the microenvironment of solid tumors (69) such as development, progression, and metastasis of tumor. The Fibroblasts can be broadly categorized into two states: quiescent and activated, despite the fact that they show substantial context-dependent phenotypic and functional variety. The cancer cells lead to the activation of fibroblasts in the TME through the release of TGFβ, PDGF, EGF, CTGF, and FGF (70). The cancer cells and CAFs are referred as ‘Partners in crime’ and inflammations support their interactions (71). These CAFs can also secrete mediators like cytokines, growth factors, CAF-specific proteins and exosomes contributing to proliferation and chemoresistance to lung cancer. The VCAM1 derived from CAFs stimulates proliferation and metastasis in A549 and H358 lung cancer cells *via* AKT and MAPK signaling (72). CAFs isolated from lung cancer tissue promote metastasis by secreting IL-6, which activates JAK2 and STAT3 signaling and also induces EMT *via* increasing vimentin expression in lung cancer cells both *in vitro* and *in vivo* (73). This CAFs leads to an immunosuppressive and growth-promoting character by modulating different immune cells in TME (74). The CAFs isolated from lung tumor patients show expression of inhibitory ligands such as PDL-2 and FASL, inhibition of these ligands can restores T cell cytotoxicity (75).

### 3.2 Endothelial cells

Endothelial and vascular blood vessels provide nutrients to tumor tissues, also serve as cancer niche cells and build an environment that supports cancer (76) *via* encouraging cancer cell migration, invasion and metastasis. They show plasticity, and can alter the cell’s fate. As the tumor progress, endothelial cells go through a process known as “endothelial-mesenchymal transition” to transform into cancer-associated fibroblasts (77). Due to hypoxic environment, these cells express various angiogenic factors like hypoxia inducible factor (HIF), vascular endothelial growth factor A (VEGFA), platelet-derived growth factor (PDGF) or angiopoietin 2 (ANGPT2) along with proangiogenic chemokines and receptors to begin neo-angiogenesis (78). These Tumor Endothelial Cells (TEC) releases cytokines which reduces the cytotoxic responses of the immune cells. The VEGF and Notch signaling pathways may be more active in TECs as compared to normal tissue endothelial cells, which is responsible for the elevation of angiogenesis (79) SIRT1 increases vessel density and negatively regulates DLL4/Notch signaling and deacetylation of Notch1 intracellular domain (N1IC) which promotes growth of Lewis lung carcinoma (LLC) xenograft derived vascular endothelial cells (80).

### 3.3 Immune cells

The important components of the tumour microenvironment are immune cells. They can either inhibit tumour growth or enhance it, depending on the circumstances, in which they interact with the tumour microenvironment. Immune cells can be broadly divided into two groups viz., innate immune cells and adaptive immune cells (77). From the available literature evidence, it is now clear that the innate immune response affects the TME by regulating T cell fate and also significantly shapes the TME. Macrophages (Ms), dendritic cells (DCs), neutrophils, myeloid derived suppressor cells (MDSCs), natural killer cells (NKs), and innate lymphoid cells (ILCs) are the innate immune cells. Mechanistically, cytokines in the TME control immunological processes, which result in suppressed immune reactions that direct tumour growth. It is crucial to gain a thorough understanding of innate immune cells and apply that knowledge to emerging treatments that intend dysregulated cells in the TME (81). Stankovic et al., 2019 has reported the presence of different immune cells in NSCLC using flow cytometry from a patient sample which were B cell, T cell, natural killer cell, macrophages, dendritic cells (82). Further the immune cells in the NSCLC microenvironment are discussed in following sections.

#### 3.3.1 Macrophages

The extraordinary plasticity of macrophages allows them to adapt their physiology in response to environmental signals, giving rise to numerous subpopulations of macrophages with diverse roles. Tumor-associated macrophages or TAMs, are found to be increased in the stromal compartment of a variety of solid tumors and orchestrate to the development, angiogenesis, and metastasis of cancer (83). In NSCLC microenvironment, TAM acts as an immunosuppressive cell and favors proliferation and metastasis of NSCLC (84). Macrophages can typically be polarized into M1 or M2 macrophages. Classically activated macrophages, often referred to as M1-polarized macrophages. TAMs are believed to more closely resemble M2-polarized macrophages, also referred to as alternatively activated macrophages, TAMs are crucial in establishing the link between inflammation and cancer (85). NOX4 is highly expressed in NSCLC cells, which recruits TAM (M2 Macrophage) through production of different cytokines that are dependent on ROS/PI3K signaling, thus promotes NSCLC growth (86). In NSCLC patients, high density of TAM is associated with poor survival and vice versa. Studies have reported that TGF‐β secreted by TAM increases SOX9 expression through C-jun/SMAD3 pathway leading to metastasis of NSCLC (87).

TAMs, which comprise the majority of immunoregulatory cells in tumors, help to suppress cytotoxic T lymphocyte (CTL) responses in tumour microenvironments. In murine tumor models, proliferation of CD8+ T cell is suppressed by TAMs, which rely on L-arginine metabolism *via* inducible nitric oxide synthase (iNOS) or arginase I, which leads to production of reactive oxygen species (ROS). TAMs also produce IL-10, which induces the expression of PD-L1 (costimulatory molecule) in monocytes, and can suppress CTL responses. Additionally, TAM derived IL-10, indoleamine 2,3-dioxygenase, prostaglandin E2 (PGE2) and CCL17, CCL18, and CCL22 results in significant roles in the activation of Tregs and inhibition of T cells respectively in the tumour microenvironment (85).

#### 3.3.2 Dendritic cell

Dendritic cells (DCs) are important for the initiation of antigen specific immune response and tolerance, as a result exploiting DC manipulation has a great potential for generating effective antitumor immunity (88). DCs are classified into three types such as conventional DCs (cDCs),plasmacytoid dendritic cells (pDC) and monocyte derived DCs (moDCs) (89). Further, conventional DCs (cDCs), are of two types’ conventional type 1 DCs (cDC1s) and conventional type 2 DCs (cDC2s). cDC1s present antigen to CD8+ T cells while cDC2s activates the CD4+ T cell response and pDC produces large amounts of interferon-α (90). DCs derived from NSCLC patients are immunosuppressive as it up regulates the expression of co-inhibitory molecule B7-H3, thus stimulating the T cell suppressive effects of DCs (91). NSCLC cells modulate the CD1c+ DC subsets functioning through regulating CD205 and CD103 expression on CD1+c DCs, which could be one aspect of NSCLC induced immunosuppressive microenvironment (92, 93). The co-culture of DCs with lung carcinoma cells (LCC) or T cells showed increased production of TGF‐β which induces generation of CD4+CD25+ FOXP3+ regulatory T (Treg) cells to suppress T cell proliferation (94).

#### 3.3.3 T regulatory cell

The key immunosuppressive cells such as regulatory T cells (Tregs) support tumour growth by impeding the effector immune response (95). In the tumour microenvironment (TME), traditional T cell can activate and differentiate the Tregs cells, which shows potent immunosuppressive role, impede antitumor immunity which in turn contributes to the growth of tumors. The T regulatory cells are divided into two types based on biological characteristics, natural Treg (nTreg) cells, which naturally develop in thymus and Inducible Treg (iTreg) cells, derived from the naive T cells present in the periphery (96). The self-antigens in thymus induces the natural Treg cells while the iTreg cells, induced by the antigens in the periphery, are generated as a result of particular cytokine in TME. Basically, T regulatory cells are the subgroup of CD4+ T cells, which are separated from other immune cells by the expression of FOXP3 (97). Tregs are prevalent in NSCLC and suggest an increased chance of recurrence of disease in the early stage and also acts as a blockade to Immune checkpoint inhibition (ICIs) in Lung cancer (98). The study has shown Foxp3+ regulatory T cells are critical for lung tumorigenesis in K-ras mutant mice model (99). Enlisted are the mechanisms through which Tregs play immunosuppressive roles. 1). Tregs releases inhibitory cytokines like TGF-β, IL-10 and IL-35 which block immune function through these and other associated pathways.Tregs can also block CD8+ T cell and DC function *via* membrane-bound TGF-β, which governs the body’s antitumor immunological function. 2). Treg cells produce granzymes and perforin which mediates cytotoxicity of effector cells. 3). They interfere with the cell metabolism, which ultimately affects function T cells by different modes like the deprivation of IL-2 (100), increasing production of adenosine *via* continuous expression CD39 and CD73 on Tregs (101). 4). Entire causes suppressive environment along MDSC (102).

#### 3.3.4 Myeloid derived suppressor cell

About 30 years ago, monocytes and neutrophils with significant immunosuppressive properties were originally described, and later given the designation myeloid-derived suppressor cells (MDSCs) to emphasize their distinct position among myeloid cells (103). MDSCs are divided into subtypes as polymorphonuclear MDSCs (PMN-MDSCs) and monocytic MDSCs (M-MDSCs) and play inhibitory role in lung cancer (104). This immunosuppressive cell steadily accumulates in tumor tissue and regulates the immune response against tumor by interacting with innate and adaptive immune cell (105). NSCLC patient study has shown endoplasmic reticulum stress in neutrophils increases the expression of Lectin-type oxidized LDL receptor-1 (LOX-1) and convert these neutrophils to suppressive PMN-MDSCs (106). Patients with non-small cell lung cancer have B7-H3 predominantly expressed on intratumoral CD14+HLA-DR/low MDSC and is associated with NSCLC progression (107). In lung cancer, MDSCs impairs the B cell response *via* inhibiting IL-7 and STAT5 signalling which is critical for the development and differentiation of B cells, therefore can promote immune escape (108).

In TME, these MDSCs are manifest to inflammatory and hypoxic surrounding, As an outcome, there is increase in arginase 1 (Arg1) and iNOS due to hypoxia-inducible factor (HIF)-1, as well as a decrease in ROS production, also increase in the inhibitory protein PD-L1 on the surface of MDSCs (109).

#### 3.3.5 Natural killer cell

NK cells were initially supposed to be big granular lymphocytes that naturally show cytotoxicity against the tumor cells. Later, it was discovered that NK cells have a distinct lymphocyte lineage that have both cytotoxic and cytokine-producing effector activities (110) but in lung cancer they are capable of relevant cytokine production rather than cytotoxicity (111). In NSCLC microenvironment, TGF-β mediates the pro-angiogenic NK phenotype *via* production of VEGF, PIGF and IFN-γ (112).

The interplay among natural killer cells and cancer cells exist, this interaction varies consistently with the development of NK cells, cancer progression and metastasis. The NK cells identify tumour cells by using a variety of surface molecule and activate them. In response to immune control, NK cells show their activity through releasing cytokines (IFN-γ, TNF-α, GM-CSF, IL-5), ADCC and also producing memory NK cells. But, the persistent exposure of NK cells with the cancer cells, alter the NK cells to immunosuppressive state through tumour derived molecules (PGE-2, PDL-1 and adenosine) and stromal cell (fibroblast, macrophage and monocyte) educated by the tumour which in turn leads to immune escape phenomena (113).

#### 3.3.6 B cell

B cells have the ability to directly present antigens to CD4+ and CD8+ T cells, shaping their ability to mount antigen-specific immune responses in the context of tumor microenvironment. In addition, depending on the constitution of the tumor microenvironment, the types of B cells present, and the antibodies they produce, tumor-infiltrating B cells may have both protumor and antitumor effects (114). In NSCLC patients, tumor-infiltrating B cells and CD4+ T cells inhabit in Tertiary lymphoid structures (TLS) and these are associated with better prognosis (115). The study has revealed tumor-infiltrated B cells subtypes and their various roles in the development of NSCLC using single-cell RNA sequencing of NSCLC patient sample (116).

#### 3.3.7. T cell

In TME, Tumor infiltrating T lymphocytes, mainly CD4+ and CD8+ T cells and their immunoregulatory cytokines define adaptive immunity. Naive CD8+ T cells engaged with antigen-presenting cells (APC) through peptide-major histocompatibility complex I (MHC I) and T-cell receptors (TCR) on T cell further co-stimulatory signals causes conversion of CD8+ T cells into cytotoxic T cell which fight against tumor and intracellular pathogens (117). The presence of PD1 and CTLA4 on T cells interact with PDL1 and CD80/CD86 on APC respectively, ultimately inhibiting T cell activation (118). Expression inhibitory receptor on T cell leads to dysfunctional state of T cells, thus abolishing immune response against cancer cells (119). In NSCLC patients, tumor infiltrating CD8+ T cells expresses inhibitory receptors such as PD-1, Tim-3, CTLA-4, LAG-3, and BTLA which are correlated to progression of disease (120). The PD-1 targeted therapies in NSCLC patients showed an increased number of Ki-67+PD-1+CD8 T cells (15). The immune cells have a multifaceted role in the nsclc microenvironment and miRNAs are crucial players in modulating biology of tumor microenvironment. The miRNAs and their role in NSCLC is extensively discussed in the below section.

## 4 MicroRNA as regulator in TME

**Figure 4:**
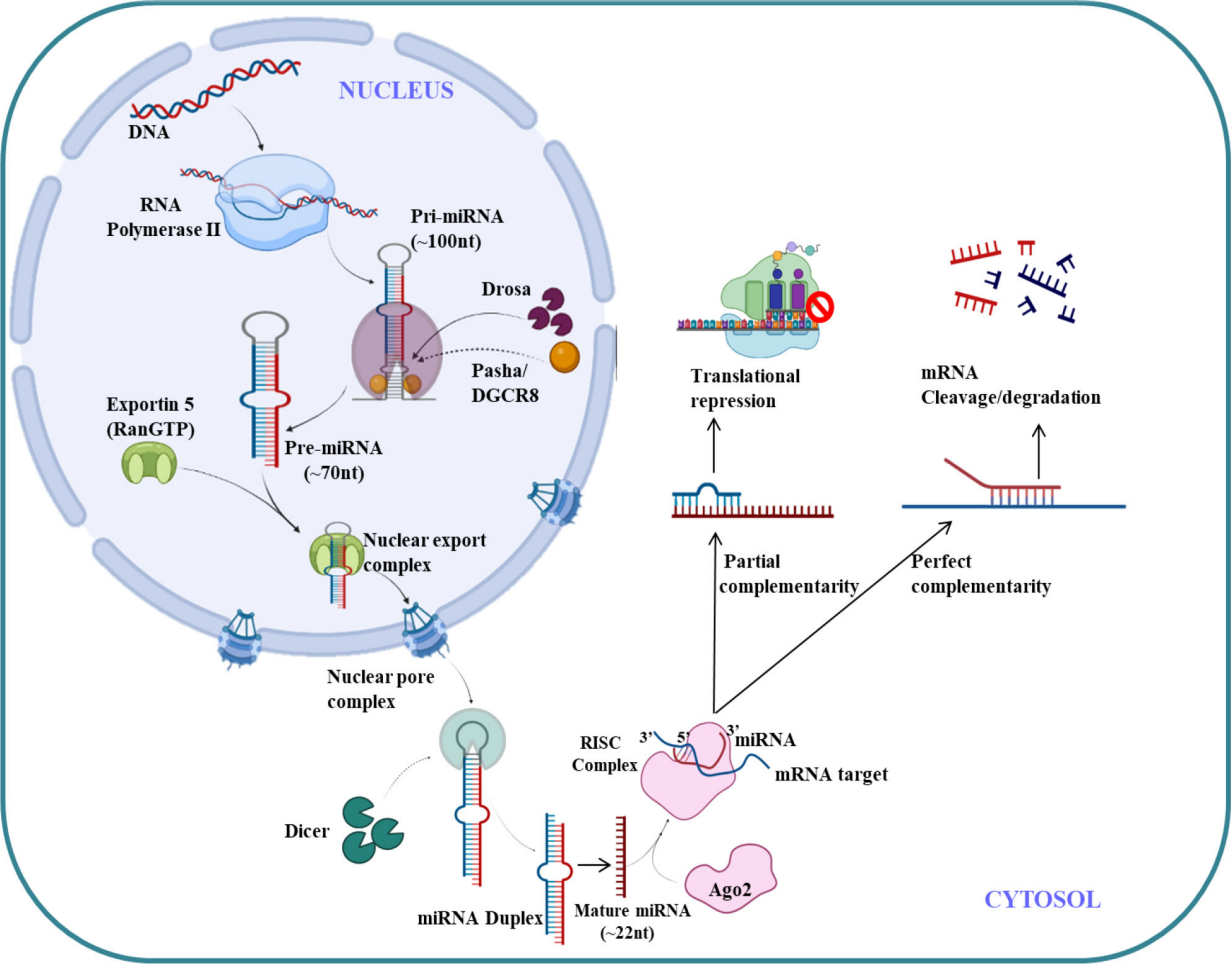
miRNA biogenesis and regulation.

MicroRNA (miRNAs) are endogenous small non-coding RNA (22 nucleotides),which regulates the gene expression (121). The first miRNA was discovered in *Caenorhabditis elegans* in 1993 which represses lin-14 mRNA (122) but it took over a decade to understand its crucial functions in gene regulation. The molecular aspects of miRNA function and biogenesis are tightly regulated at several levels and have been extensively studied (123). Canonical pathway is a dominant pathway for miRNA biogenesis. The pri-miRNA transcript is produced by RNA polymerase II/III. This pri-miRNA is cleaved by a microprocessor complex which consists of DGCR8 and Drosha to form pre-miRNA. Further pre-miRNA exported from nucleus to cytoplasm with the help of exportin 5 (XPO5)/RanGTP complex and again cleaved by Dicer to form mature miRNA duplex (124). The RNA-induced silencing complex (RISC) consist of mature miRNAs and Argonaute (Ago) proteins, which further regulates post-transcriptional gene silencing by inducing messenger RNA (mRNA) degradation or translational inhibition (125). The canonical pathway for biogenesis of miRNA is shown in **Figure 4**. The complementary sequence between the seed sequence of each unique miRNA and the target mRNAs determines the specification of target mRNA (126).

In 2002, Calin et al. were the first to uncover a linkage between miRNA dysregulation and cancer (127). Alterations in miRNA are entailed in cancer initiation and progression. The differential expression of miRNA genes in cancer and normal cells are due to the presence of these genes in cancer associated genomic regions (128). In 2006, Volinia et al. reported miRNA signature in solid cancer and validated the predicted targets of differentially expressed miRNAs as a classic oncogenes or tumor suppressors (129), as the miRNAs regulate gene expression at post transcriptional level, they have a significant impact on a broad range of pathways, however their influence is more on developmental and oncogenic pathways (128, 130). The different mechanism for dysregulation of miRNA in cancer includes various aspects such as amplification or deletion of miRNA genes, transcription factors controls miRNA expression, the dysregulated epigenetic modulation of miRNA and lastly, aberrant expression of any enzymes of miRNA biogenesis pathway could cause abnormal expression of miRNA (131).

Based on the important role that miRNAs play in lung cancer pathogenesis, they are considered to be prospective prognostic markers. There are mounting evidences which suggests that miRNAs are dysregulated in human cancer including non-small cell lung cancer (NSCLC), and may act as oncogene or tumor suppressor miRNA (132). Takamizawa et al. were the first to report the reduced expression of let-7 in lung cancer and also showed *in vitro* overexpression of let-7 in A549 cell line inhibited growth of lung cancer cells (133).

The abnormal expression of miRNAs affects the different hallmarks of cancer (131). In the TME, miRNA are supposed to connect the tumor cells and immune cells in the surrounding, thus, the dysregulated expression of miRNAs in cancer cell can directly have an impact on the immune microenvironment. For instance, miRNA can direct the production of chemokines or cytokines by tumor cells, which in turn influences immune cells. However, exosomes are crucial for miRNA transport, and can also have an impact on the immunological microenvironment since miRNA of tumor cells can be transferred *via* exosomes to immune cells (134).

## 5 miRNAs involved in EGFR signaling cascade

**Figure 5:**
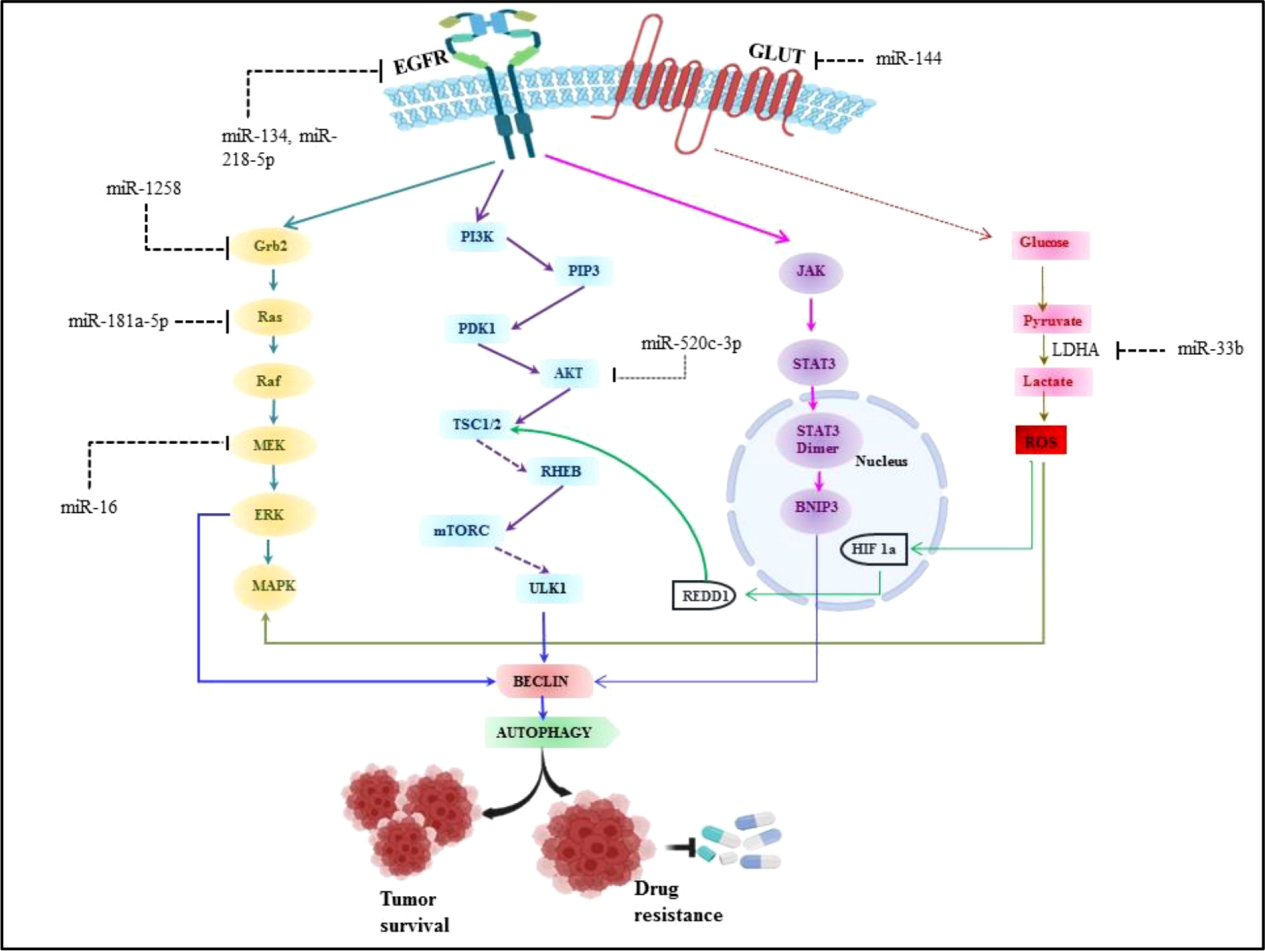
Perturbed EGFR signaling leading towards autophagy and key regulatory miRNA in EGFR signalling in NSCLC.

There are extensive reports which suggest the role of miRNA in angiogenesis, proliferation, cell metabolism and modulating immune cell response in non-small cell lung cancer. Aside from various environmental parameters, it is important to understand the molecular abnormalities in lung cancer development as the cancer cannot grow without involvement of any molecular abnormalities. Although, a number of signaling axes have been implicated in the pathogenesis of lung cancer, it seems that the fatality is due to the overlap of oncogenic pathways (135). Thus the studies have been done to investigate the various signaling axes which led to the pathogenesis in lung cancer. It has been reported in earlier studies that overexpression of Epidermal growth factor receptor (EGFR) is found in 50% of NSCLC and it is associated with poor prognosis (136). The majority of EGFR kinase domain mutations are found at exons 18 to 21, which increases EGFR’s kinase activity and triggers over activation of downstream signaling pathways, the three major downstream signaling pathways such as phosphatidylinositol 3-kinase (PI3K)/Akt/mTOR, interleukin 6(IL-6)/Janus kinase (JAK)/signal transducer and activator of transcription 3 (STAT3), mitogen-activated protein kinases (MAPK)/extracellular signal-regulated kinases (ERK) which supports tumorigenesis of NSCLC (3, 136). Approximately 90% of all EGFR mutations are of the two types, L858R (point mutations in exon 21 that result in a leucine to arginine substitution at codon 858) and exon 19 deletion (in-frame deletions in exon 19) (137). miRNA expression patterns that are abnormal can be employed as prognostic and diagnostic indicators in NSCLC. The development of NSCLC is strongly influenced by EGFR signaling pathways and miRNAs. Numerous studies have demonstrated the dual function of miRNAs as either oncogenes or oncosuppressors, and that it primarily depends on their direct targets and the activated downstream signaling pathways (138, 139). It was discovered that EGFR, MET, and the miRNA cluster 23a27a24-2 were biologically connected, with miR-27a controlling MET, EGFR, and Sprouty2 in a variety of NSCLC cell lines. These findings suggest that miR-27a can inhibit MET and EGFR by directly or indirectly targeting their 3’ UTRs *via* Sprouty2 (140). Luciferase assays revealed that EGFR was a direct target of miR-134, and its overexpression in NSCLC cells inhibited EGFR expression (141, 142). Another miR-218-5p also inhibits EGFR in A549 and H1975 cell lines (143). To determine the miRNA-dependent EGFR signaling cascade, database-dependent miRNA target prediction was carried out. This model demonstrated that the circulating miR-145, miR-199a-5p, and miR-495 have favorable correlations with the EGFR ligand EGF. It follows that miRNAs can control several EGFR signaling pathway elements, including the ligand, receptor, or downstream signaling molecules (144, 145). According to findings, mutant K-Ras negatively affects miR-199b expression in NSCLC cells by overexpressing it and silencing it. Overexpressing mutant K-Ras inhibits miR-199b expression, while silencing it increases it. MiR-199b functions as an anticancer agent by concurrently blocking the Akt and ERK pathways by specifically targeting numerous Akt and ERK pathway components in NSCLC (146). Our previous system biology study reported miR-520c-3p could target AKT signaling in NSCLC (147). Downregulated miR-16 involved in lung cancer proliferation and invasion through ERK/MAPK signaling which inhibits MEK1 (148). miR-1258 inhibits NSCLC proliferation *via* downregulating GRB2 expression both *in vitro* and *in vivo* (149). Similarly miR-181a-5p downregulates Kras in A549 cells, thus inhibiting NSCLC proliferation (150). miRNAs are also known for their regulatory role in NSCLC metabolism.miR-33b also affects the glucose metabolism in NSCLC by downregulating LDHA enzyme (151) and miR-144 targets GLUT1 thus involved in Warburg effect in lung cancer (152). Perturbed EGFR signaling leading towards autophagy and key regulatory miRNA in EGFR signaling in NSCLC is represented in **Figure 5**.

Furthermore, miR-224 promotes tumor growth in NSCLC both *in vitro* and *in vivo* by directly targeting the tumor suppressors TNFAIP1 and SMAD4 (153). Specifically miR-218 downregulate the IL-6 receptor and JAK3, thus, by overexpression miR-218 in NSCLC cells prevents cell proliferation, invasion, and colony formation (154). miR-196b-5p downregulates the tumor suppressors, GATA6 and TSPAN12 expression. Report suggested that TSPAN12 knockdown H1299/shTSPAN12 cells were injected in nude mice, resulting in significantly increased tumor size (155). Additional research revealed that miR-7-5p inhibits NSCLC tumor metastasis by targeting NOVA2. miR-7-5p mimic and negative control was injected in the A549 xenograft model, leading to decrease in tumor growth and metastasis. while NOVA2 overexpression reduces the inhibitory effect of miR-7-5p on lung cancer cells (156). Similarly, miR-187-5p downregulates CYP1B1 which is involved in the development and metastasis of NSCLC cells (157).

## 6 miRNA as immunomodulator in NSCLC microenvironment

**Figure 6:**
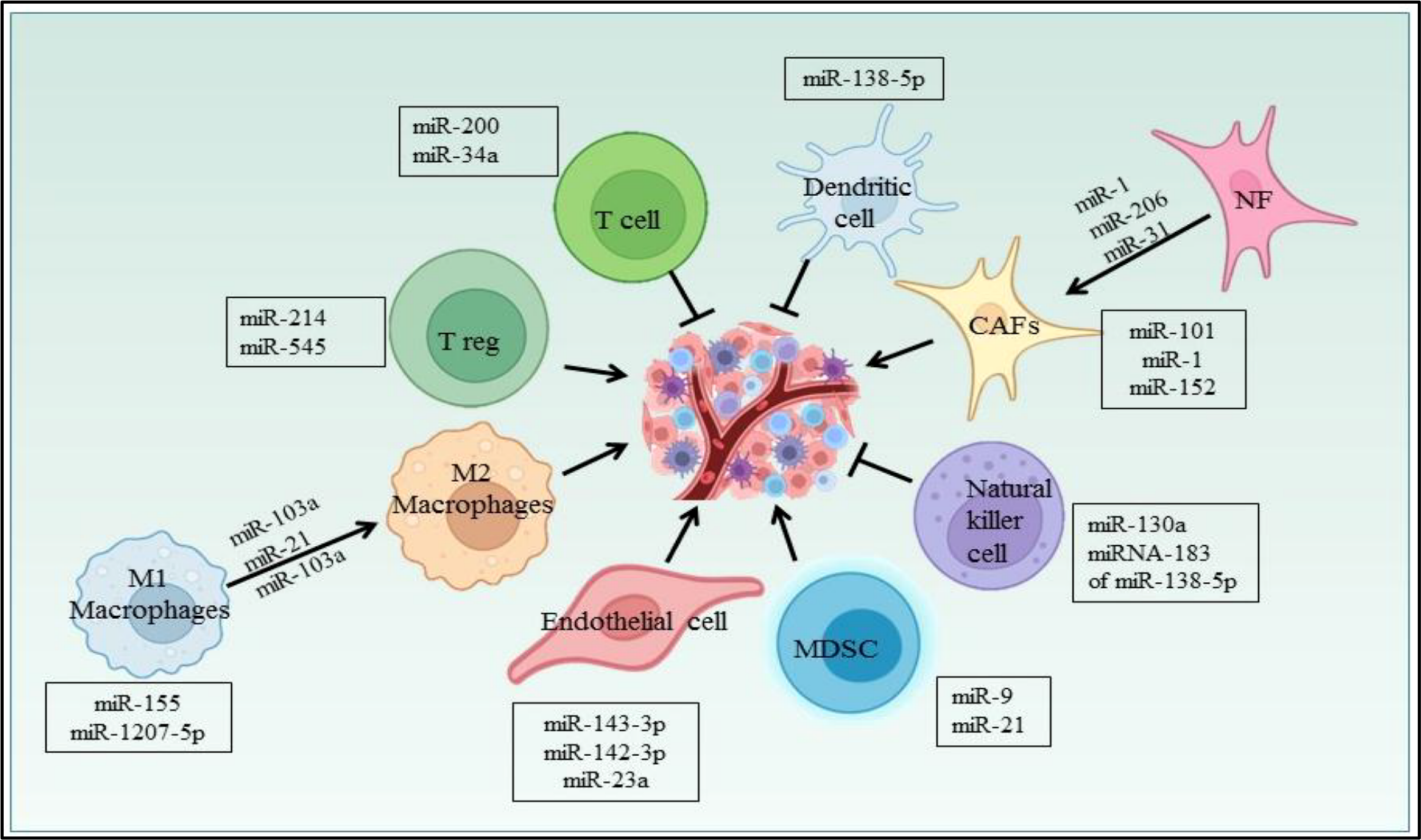
miRNA regulating different cells in the NSCLC microenvironment.

Several miRNAs have been reported to regulate the cells in the NSCLC microenvironment (**Figure 6**). CAFs, being one of them, contributing towards proliferation and drug resistance in NSCLC *via* miR-1. miR-1 mediates SDF-1 expression, which upregulates CXCR4 expression, leading to increased expression of NF-κB and Bcl-xL in A549 and 95D lung cancer cells (158). The study has shown miRNAs plays role in conversion of normal fibroblast in CAFs in NSCLC patient, with this aspect miR-1, miR-206 were down regulated and miR-31 was upregulated in CAFs of Lung regulating FOXO3a/VEGF/CCL2 signaling (159). Moreover miR-101 is downregulated in CAFs of lung cancer which targets the CXCL12 expression thus overexpression of this miRNA could suppress the CAFs to promote cancer cell proliferation (160). miR-21 and miR-103a involved in stimulating the polarization of M1 to M2 phenotype in lung cancer, also activates AKT-STAT3 signaling axis while tumor suppressor miR-1207-5p and miR-155 stimulates M1 polarization (161). The previous study also suggests that extracellular vesicles derived from hypoxia lung cancer causes increased M2 polarization *via* transfer of miR-103a. This miR-103a decreases PTEN level, ultimately increasing activation of AKT and STAT3, also expressing many immunosuppressive and pro angiogenic factors (162). miR-20a down regulates the expression PTEN and up regulates PDL1 expression thus leading to proliferation of NSCLC (163). The Natural killer cells isolated from patients with NSCLC showed reduced expression of miR-130a and elevated expression of STAT3. miR-130a can target STAT3, thus overexpression of miR-130a enhances NK-92 cells’ killing activity against A549 cells (164). TGF-β is a potent immunosuppressive found in the NSCLC microenvironment and study reported that TGF-β induced miRNA-183 expression represses DAP12 in NK cells affecting cytotoxic response of NK cell against tumor cell (165). Nannan Song et al. have reported the role of miR-138-5p in NSCLC. This miRNA downregulates CCND3, Ki67, CCD20, MCM in A549/3LL cells and is also found to regulate DCs maturation in nude and C57BL/6 mouse models bearing A549 and 3LL cells respectively. Furthermore miR-138-5p downregulates PDL1 in A549 and PD1 on DCs (166).

In lung cancer, miR-21 regulates MDSCs accumulation and activity. miR-21 downregulates RUNX1 and upregulates YAP in lewis lung cancer mice model promoting immunosuppressive role of MDSC (167). Also miR-9 regulates differentiation and function of MDSC *via* targeting RUNX1. miR-9 downregulation in MDSCs inhibits *in vivo* tumor growth in lewis lung cancer mouse model (168). Yuan Yin et al. showed LLC cells secretes miR-214 which targets PTEN in recipient CD4+ T cells results in promotion of Treg expansion (169). The radiotherapy treatment on lewis lung cancer tumor inhibits Treg recruitment *via* increasing miR-545 expression (170). miR-34a mimic MRX34, the first miRNA based therapeutic which entered in first phase clinical trial as an anticancer drug (171). The studies also suggest the miR-34a mimic can modulate the tumor infiltrating immune cells. In NSCLC mouse model, specifically it was shown that *in vivo* injection of MRX34 generates the lower expression of PD-L1, but MRX34 can also boost tumor infiltration of CD8+ T cells and reduce CD8+PD1+ T cells (172). miR-152 downregulates fibroblast growth factor 2 (FGF2) which in turn suppresses NSCLC proliferation (173). The study showed PD-L1 expression is regulated by the miR-200/ZEB1 axis in NSCLC (174). Previously reported that miR-34a, tumor suppressor downregulates CDK6 expression and high expression of miR-34a is associated with a prolonged progression-free survival in NSCLC patients (175). miRNAs also play a role in NSCLC angiogenesis. miR-519c targets HIF-1α which is an angiogenesis regulator (176). In hypoxia, lung cancer cells secretes exosomes, in which miR-23a is upregulated and targets PHD1 and 2 contributing to accumulation of HIF-1α,in turn to angiogenesis (177).

## 7 Autophagy

Autophagy is an intracellular catabolic degradation process in which cytoplasmic macromolecules, aggregated proteins, damaged organelles, or pathogens are delivered to lysosomes and digested by lysosomal hydrolases to generate nucleotides, amino acids, fatty acids, sugars, and ATP before being recycled into the cytosol. Eliminating aggregated proteins and damaged organelles, autophagy is a highly conserved catabolic cellular event. The dynamic process entails induction, autophagosome nucleation, development of the double membrane, sealing and fusion with the lysosome, and breakdown of absorbed components. In order to degrade and recycle non-functional cellular components as an internal source of nutrition, cells typically have basal levels of autophagy. The amount of autophagy may significantly increase in response to a variety of stressors and stresses, including deprivation, hypoxia, and medication, in order to supply intracellular nutrition and remove toxic components (178, 179). Initiation, vesicle nucleation, vesicle maturation, vesicle fusion, and cargo degradation are the five steps of the autophagy pathway. It starts with the activation of the Unc-51-like kinase 1 (ULK1) complex, which includes ULK1, ULK2, autophagy-related gene 13 (ATG13), focal adhesion kinase interacting protein 200 kDa (FIP200), and ATG101. Activated ULK1 kinase, mediates the phosphorylation and activation of the Beclin1-VPS34 (a class III phosphatidylinositol 3-kinase (PI3K)) complex and eventually instigate vesicle formation. Various protein conjugation events are required during the maturation process to help cleave pro-LC3 to soluble LC3-I, which is then conjugated to lipid phosphatidylethanolamine (PE) on the surface of the emerging autophagosome. After being conjugated to lipid, LC3-I is inserted onto the surface of emerging autophagic vesicles. The autophagosome is what happens when the isolation membrane is enclosed, and is then transported to the perinuclear region where the lysosome subsists. Here certain membrane-tethering complexes, SNAREs and Syntaxins aid in autophagosome-lysosome fusion. Finally, after the fusion lysosomal hydrolases degrade autophagic cargo, and recycled contents are discharged *via* nutrient transporters, fueling cell growth (180).

### 7.1. Environmental based accountabilities of autophagy in NSCLC

It appears that there are two possible outcomes for the role of autophagy in tumorigenesis. It appears to play a role in tumour suppression in the early stages of cancer (181, 182), and in later stages, it allows cancer cells to survive metabolic stress and hypoxia in solid tumors (183). According to a research by Rao et al. in a mouse model of non-small cell lung carcinogenesis they concluded that autophagy plays a dual role in oncogenesis. Cancer cells need to be in the best possible shape to withstand a hostile environment caused by hypoxia, as well as the lack of nutrients and trophic stimuli, particularly at advanced stages of tumour progression. However, autophagy is frequently inhibited during early oncogenesis, raising the possibility that autophagy is an oncosuppressive mechanism (184). The environment does affect autophagy’s function in cancer, and its upregulation is required for cancer cells to survive in hypoxic tumour regions (185).

### 7.2 Autophagy modifies the NSCLC tumor microenvironment

Literature evidence suggests that the tumor microenvironment is affected by autophagy. TME consists of several factors like hypoxia, inflammation, and cytokines. Under stressful situations in cancer microenvironments, autophagy meets the need for cellular energy and avoids cytotoxicity. Cancer cells could be able to adapt and survive in low oxygen environments by using the stress response when hypoxic circumstances are present. TME uses autophagy to provide the metabolic requirements of cancer stem cells, sound immune cells, cancer associated fibroblasts, angiogenesis, neural connections, and extracellular matrix (186).

Autophagy is proposed as a cell death mechanism, programmed cell death type II, and has been shown to play a greater variety of pathophysiological roles in many disease processes, including cancer; it also aids cells to clear damaged proteins, organelles, pathogens, or aggregates. Autophagy’s potential to modulate cell death makes it a therapeutic target in cancer (179). By controlling their target genes, microRNAs (miRNAs) were frequently shown to be dysregulated in NSCLC and were strongly linked to the formation, progression, and metastasis of the disease.

## 8 miRNA and autophagy

In addition to genetic factors, epigenetic mechanisms like microRNA networks are crucial for controlling autophagy-related pathways. Numerous miRNAs can alter autophagy and the processes that depend on it in a number of disorders. More studies have shown a connection between autophagy-regulating miRNAs and chemoresistance/chemosensitivity in lung cancer cells (187, 188). As previously mentioned, mTORC1 is inhibited in response to a variety of stress-inducing signals, which activates ULK1/2 and initiates autophagy. It has been demonstrated that miR-520a-3p, miR-513b, and miR-145 alter the mTOR signaling cascade to reduce NSCLC cell proliferation and migration (189, 190). In NSCLC tissue,miR-21 is highly expressed and regulates autophagy in A549 cells through AMPK/ULK1 signaling pathway (191). By directly downregulating HMGB1 and subsequently activating the PI3K/Akt/mTOR pathway, miR-142-3p overexpression prevents starvation-induced autophagy of NSCLC cells. Additionally, miR-142-3p overexpression decreases autophagy triggered by anticancer drugs and raises the chemosensitivity of NSCLC *in vitro* and *in vivo* (192). Studies indicate that microRNAs and treatment resistance in NSCLC patients are closely related. In a study, it was discovered that patients with gefitinib-resistant NSCLC have decreased expression of miRNA-153-3p. The downregulation of ATG5 caused by the overexpression of miRNA-153-3p decreased autophagy and increased gefitinib sensitivity in NSCLC (193).

## 9 Therapeutic roadmap

The last ten years have seen a significant advancement in NSCLC therapy, allowing for a more tailored choice of alternatives. Treatment options are influenced by immunologic and molecular characteristics. Patients with EGFR mutations, ALK rearrangements, ROS1 rearrangements, BRAF mutations, NTRK mutations, and high levels of PD-L1 for instance, should receive FDA-approved targeted therapy (**Table 1**) or immunotherapy as their first line of treatment. RET, MET, and HER2 in NSCLC are three additional oncogenic driver mutations that are intriguing therapeutic targets. The immune system’s ability to block PD-1, PD-L1, and CTLA-4 is also expanding (194).

**Table 1:** Available targeted therapies.

Target	Available drug candidate	References
**EGFR-TKIs**	Gefitinib(1^st^ Gen), Erlotinb(1^st^ Gen), Afatinib(2^nd^ Gen), Dacomitinib(2^nd^ Gen) Osimertinib(3^rd^ Gen), · L858R, ex19del and T790M resistant mutants Nazartinib (EGF816) (3^rd^ Gen), BPI-7711(3^rd^ Gen), Lazertinib (YH25448) (3^rd^ Gen),HS-10296(3^rd^ Gen)	(MAURICIO BUROTTO et al,. 2015), (Ramalingam, Suresh S et al,. 2019), (David Konig et al,. 2021)
**ALK fusion/rearrangement inhibitors**	Crizotinib(1^st^ Gen), Ceritinib(2^nd^ Gen), Alectinib(2^nd^ Gen), Brigatinib(2^nd^ Gen), Lorlatinib(3^rd^ Gen),	(Sacha I. Rothschild et al,. 2013) (Fabrice Branle et al,. 2017) (Byoung Chul Cho et al,. 2019),
**ROS1/NRTK inhibitor**	Crizotinib, Larotrectinib, Entrectinib, DS-6051b, Repotrectinib	Jessica J.Lin and Alice T.Shaw,2017)(Meng Liu et al., 2022)(Anna F.Farago et al., 2017)(Anna M. et al., 2022)(Bo Mi Ku et al., 2020)
**BRAF V600E mutations**	Dabrafenib and Trametinib	(Jean G and Gregory A2019)(Ninging Yan et al., 2022),
**MET inhibitors**	Crizotinib, Cabozantinib, Capmatinib, Tepotinib	(Sukhmani Padda et al., 2012) (Mariacarmrla S.et al 2021)(Qiming Wang et al., 2019)
**RET fusion/rearrangement inhibitors**	Cabozantinib, Vandetanib, Lenvatinib, sunitinib, RXDX-105, BLU-667, LOXO-292,	(Priscilla Cascetta et al., 2021)(Thomas E 2020)(Noura J. Chaudhary and Alexander Drilon 2020)
**HER2**	Trastuzumab deruxtecan, XMT-1522, Poziotinib	(M. Riudavets et al., 2021)(Bob T et al., 2022)
**KRAS G12C inhibitor**	AMG510, MRTX849	(Gabriela Palma et al., 2021)(Juan Bautista B. 2021)
**ATR inhibitor**	M6620	(Thomas Anish et al., 2021)(Mark R.Middleton et al. 20 2021)
**AXL kinase inhibitor**	TP-0903, Bemcentinib	(Sun Min Lim et al., 2022) (Chenjing Zhu et al., 2019)

### 9.1 Targeted therapies

#### 9.1.1 Epidermal growth factor receptor tyrosine kinase inhibitors

EGFR is a member of the receptor tyrosine kinase family, which also includes HER2/ERBB2/NEU, HER3/ERBB3, and HER4/ERBB4. EGFR mutations are more prevalent in never smokers. These mutations affect EGFR exons 18–21, and 90% of them are exon 19 deletions or exon 21 L858R point mutations. Gefitinib is the first EGFR Tyrosine Kinase inhibitor investigated for use in advanced NSCLC. At present, FDA has approved the use of gefitinib, erlotinib, afatinib, dacomitinib, and osimertinib as first-line treatments for patients with metastatic NSCLC (195–197).

#### 9.1.2 ALK fusion/rearrangement inhibitors

About 2-7% of patients with advanced NSCLC carry the fusion oncogene EML4-ALK; these patients are often younger and never smokers. The first recognised targeted treatment for ALK-positive advanced NSCLC is crizotinib. Alectinib is currently the first-line therapy of choice. Another next-generation ALK inhibitor, brittinib, outperformed crizotinib in terms of Progression free survival (198). A potent ALK TKI, Ensartinib (X-396) has also demonstrated inhibitory efficacy against MET, BAL, Axl, EPHA2, LTK, ROS1, and SLK (199).

#### 9.1.3 ROS1/NRTK inhibitor

About 1-2% of NSCLC cases involve ROS1 rearrangement, which mainly affects younger people who have never smoked. The tyrosine kinase receptor is encoded for by the ROS1 gene, which is located on chromosome 6 (198). NTRK1 gene fusions can also involve NTRK2 or NTRK3 and is present in all forms of tumors. Such fusions represent roughly 1% of NSCLC (200). For metastatic ROS1 positive NSCLC, crizotinib received approval in March 2016. For ROS1, ALK, and NTRK gene fusions, entrectinib is an oral TKI. The use of entrectinib for the treatment of advanced or recurrent NSCLC with NTRK fusions in adults and children was first authorized in Japan in June 2019. Recently, the FDA approved entrectinib for use in both these indications and for advanced NSCLC patients who test positive for ROS1 (200).

#### 9.1.4 BRAF V600E mutations

1-3% of NSCLC patients with a linked history of smoking had an activating BRAF mutation. Based on an open-label trial, the combination of dabrafenib and trametinib was approved for the treatment of metastatic NSCLC with BRAF V600 E mutations (201).

#### 9.1.5 MET inhibitors

Hepatocyte growth factor binds to the tyrosine kinase receptor MET. Characteristic anomalies that result in enhanced MET signalling activity include MET gene amplification and exon 14-skipping mutations. Only 3% of instances of NSCLC had an isolated MET exon 14 mutation, however 15-20% of EGFR mutation-positive NSCLC patients have this acquired EGFR TKI resistance pathway instead. Highly selective MET inhibitors include capmatinib and tepotinib (202, 203).

#### 9.1.6 RET fusion/rearrangement inhibitors

The RET rearrangement leads in ligand independent homodimerization and activation of the RET tyrosine kinase by autophosphorylation. It is composed of the RET intracellular kinase domain and the coiled coil domain of the partner gene. As a result, the phosphoinositide 3-kinases (PI3K)/AKT, mitogen-activated protein kinase (MAPK), and signal transducer and activator of transcription 3 (STAT3) pathways are activated, increasing cell proliferation, survival, migration, and differentiation (204). Experiments have been performed on multi-targeted TKIs (MKIs), including cabozantinib, vandetanib, lenvatinib, and sunitinib, which target RET fusion-driven NSCLCs. The most investigated RET inhibitors include cabozantinib and vandetanib, both of which exhibited a response rate in patients with RET-rearranged NSCLC between 20 and 50% (205).

#### 9.1.7 HER2 inhibitors

2-4% of NSCLC patients have HER2 alterations, which primarily show up as protein overexpression, gene amplification, or gene mutation. Female, never-smoker, and lung adenocarcinoma patients with HER2 modifications had a higher risk of developing brain metastases than individuals without HER2 abnormalities or other genetic mutations (206). Pyrotinib is an oral pan-TKI that blocks HER1, HER2, and HER4 (108).

#### 9.1.8 KRAS G12C inhibitor

One of the most frequent oncologic driver mutations in advanced NSCLC is the KRAS mutation. About 14% of lung adenocarcinomas have the KRAS G12C mutation. The median survival is reduced in NSCLC patients who also carry KRAS. AMG510 is a small, promising molecule that locks KRAS G12C in an inactive GDP-bound state, specifically inhibiting it. Another KRAS G12C inhibitor, MRTX844, is effective in treating advanced solid tumours with KRAS G12c mutations (207, 208).

#### 9.1.9 ATR inhibitor

Inhibiting ATR signaling may make tumors more sensitive to chemotherapy that damages DNA because ATR is a crucial part of the DNA damage response. ATR is specifically inhibited by M6620. When combined with gemcitabine, berzosertib is well tolerated by patients with advanced solid tumors (209).

#### 9.1.10 AXL kinase inhibitor

AXL is a receptor tyrosine kinase from the TAM family, together with MER and TYRO3. Despite the fact that AXL itself is not regarded to be a very strong oncogenic driver, overexpression of AXL has been shown to promote tumour cell growth, survival, invasion, metastasis, angiogenesis, epithelial to mesenchymal transition, and immune suppression. Bemcentinib is a very specific inhibitor of the AXL kinase (210).

### 9.2 Antibody-drug conjugates

Despite the fact that both targeted therapy and immunotherapy-based approaches have become the frontline gold standard of care for patients with advanced lung cancer, acquired resistance and disease progression are nevertheless frequently unavoidable. In this situation, chemotherapy is frequently used as a last resort, but it has a somewhat limited therapeutic index. The development of antibody-drug conjugates (ADCs) presents an enticing substitute. ADCs enable the targeted delivery of cytotoxic payloads to cancer cells by fusing the specificity of a monoclonal antibody with the cytotoxic effects of chemotherapy. Three essential parts make up an ADC construct: the payload, the linker, and the antibody. Each component of an ADC must be carefully created and engineered in order to produce a stable molecule that strikes a balance between efficacy and toxicity (211, 212). Ado-Trastuzumab Emtansine (T-DM1) is an ADC construct made of trastuzumab, an anti-HER2 monoclonal antibody, and emtansine (DM1), a microtubule inhibitor (213). A new ADC called trastuzumab Deruxtecan (T-DXd) combines trastuzumab, a humanized anti-HER2 monoclonal antibody, with deruxtecan, a topoisomerase inhibitor, through a protease-cleavable peptide linker (214). Trastuzumab, which is HER2-targeted, is coupled to the monomethyl Aurstatin F derivative duostatin-5 by a protease-cleavable valine citrulline linker in the drug A166 (139). A glycoprotein trans-membrane calcium signal transducer known as Trophoblast Cell-Surface Antigen (Trop-2) is thought to be a mediator of cell migration and anchorage-independent growth. A number of epithelial malignancies, including NSCLC and small cell lung cancer (SCLC), have been found to express it (215). The anti-Trop-2 monoclonal antibody sacituzumab is connected to the topoisomerase I inhibitor SN-38 *via* a hydrolysable, cleavable linker to form the anti-drug conjugate sacituzumab govitecan (216). A humanized anti-Trop2 mAb, a tetrapeptide-based cleavable linker, and the exatecan derivative DXd make up the drug datopotamab-deruxtecan (217). To fully utilize the capabilities of the upcoming ADC generation, numerous additional tactics are being investigated. ADCs with immunotherapy are the most alluring combo to encourage synergy.

### 9.3 Immunotherapy

Cancer immunotherapy functions by enabling our own immune systems to combat the disease. The immune system can be stimulated to target cancer cells by vaccination therapy (active immunotherapy) or immune blockade removal (passive immunotherapy) using checkpoint inhibitors. Nivolumab, an immune checkpoint inhibitor, was the first immunotherapy for metastatic NSCLC that was approved and is made entirely of human IgG4. It binds to the PD-1 receptor and prevents the tumour PD-L1 from attaching to the T cell PD-1 receptor, restoring anti-tumor immunity. Inhibitors of the PD-1 immune checkpoint include nivolumab and pembrolizumab. A fully humanized IgG1 anti-PD-L1 antibody is atezolizumab (218). Atezolizumab has just received approval for the first-line therapy of adult patients with metastatic non-squamous NSCLC who do not have EGFR or ALK genetic tumour abnormalities. It is used in conjunction with bevacizumab, paclitaxel, and carboplatin (219). Regardless of PD-L1 expression, first-line treatment with nivolumab with ipilimumab in advanced NSCLC patients exhibited better overall survival compared to individuals treated with chemotherapy. In patients with solid malignancies, including NSCLC, tislelizumab (anti-PD-1) exhibited evidence of anti-tumor effectiveness and was well tolerated. In addition to anti-PD-1/PD-L1 monotherapy, combination immunotherapy has also demonstrated effectiveness in patients with high PD-L1 expression (220). An immunoglobulin-like protein called Siglec-15 may be overexpressed in a variety of human malignancies. PD-L1 and siglec-15 expression are mutually exclusive and function as important immune suppressors. Monoclonal antibody NC 318 targets Siglec-15 to restore immune system normalcy (35). It has been proposed that interleukin 8 (IL-8) promotes immunological evasion. A human monoclonal antibody called BMS-986253 binds to and prevents the action of IL-8. A study revealed that BMS-986253 monotherapy was well tolerated in solid tumors with metastatic or irresectable disease (221). TNF receptor CD40 is a pro-inflammatory molecule. An antigen-presenting cell is activated by the humanized monoclonal antibody APX005M binding to CD40, which prompts the activation of T cells that are specifically tuned to respond to malignancy (222). Although recent developments in immunotherapy have been encouraging, additional pre-clinical and clinical research with an immunotherapy focus is needed to enhance patient clinical prognosis. Immunotherapy is already being used to treat NSCLC in certain circumstances.

Growing interest in modified-T-cell treatment, particularly that utilizing chimeric antigen receptor (CAR)-T cells, has been observed recently in a variety of solid malignancies. Clinical trials have shown promising outcomes, and the US Food and Drug Administration has authorized the use of anti-CD19 CAR-T cells for the treatment of hematological B-cell malignancies. Recent developments suggest that CAR-T cell therapy is also an effective method for treating NSCLC (223). But, T cell exhaustion is a condition that affects T cells that infiltrate lung tumors. According to a recent study, the NR4A transcription factor family was proven to have a significant impact on T cell exhaustion and to restrict CAR-T cell activity in solid tumors. They showed that CAR-T cells work better in mouse models without NR4A transcription factors, resulting in smaller tumors and higher survival rates (224).

Engineered immune-cell-based cancer treatments have shown strong success in B cell malignancies, but obstacles such as the scarcity of optimal targetable tumour antigens, tumor-mediated immunosuppression, and significant toxicity continue to limit their therapeutic efficacy and widespread use. By using synthetic biology, these difficulties can be solved and more powerful, effective adaptive medicines can be developed, allowing for the precise targeting of cancer cells while preserving healthy cells (225).

## 10 Synthetic biology as therapeutic approach for NSCLC

Synthetic biology pertains to engineering fundamentals which intend to amend living cells, permitting them to execute functions defined by the user and to obtain desired results (225). This field reconciles engineers and biologists to rewire and reprogram in organisms, through designing constructs for biomolecular components, pathways and networks (226). In 1980, Barbara Hobom introduced synthetic biology to define genetically engineered bacteria by recombinant DNA technology. Meanwhile in 2000, Eric Kool and other spokesperson reintroduced the term ‘synthetic biology’ at American Chemical Society’s yearly meeting which was held in San Francisco. Here, the name was used to define the synthesis of synthetic organic molecules that show role in living systems. Broadly, the word also has been used as ‘redesign life’ (227). The different scientific committee in world define the synthetic biology but there is no approved worldwide definition, the main characteristics of synthetic biology comprises “the *de novo* synthesis of genetic material and engineering-based method to develop components, organism and products” advocates by the report of the Secretariat of the Convention on Biological Diversity (2015) (228).

Synthetic biology’s only aim is to contribute towards the development of biomedical breakthroughs including escalating bacterial resistance to antibiotics, the rapid rise of novel infectious diseases and the emerging resistance to cancer drugs. Thus to solve these issues, synthetic biology anticipates the construction of specifically designed, easily controlled, and secure devices that would support human immune systems and correct metabolic anomalies (229). The first reports of genetic circuits that had been built to perform certain functions were reported in January 2000. Here, Collins and colleagues constructed a genetic toggle switch which is a synthetic, bistable gene-regulatory network in *Escherichia coli* and offered a straight forward theory that forecasts the conditions obligatory for bistability. The designed toggle switch comprises two constitutive promoters and two repressors, where each promoter regulates the expression of repressors that are antagonistic to one another (230). Researchers have created fundamental components and elements that allowed transcriptional, translational, and post-translational control of biological processes during the “first wave” of synthetic biology (231). It has exploded in the last ten years and is poised to revolutionize biotechnology and medicine (232). Synthetic biologists endeavor for useful qualities to optimize gene circuits like modularity, orthogonality, tunability, composability. The Design-build-test-learn optimization may be needed before the desired function is fulfilled, as the circuit behaviour rely on the specific cellular context (233). A research group has reported computational and experimental methods to characterize the circuits depending on growth factor exchange in two cell types such as macrophage and fibroblast, and discovered that the circuit is steady and resistant to perturbation (234). Many approaches with the use of synthetic biology have been investigated for treating cancer.

**Figure 7:**
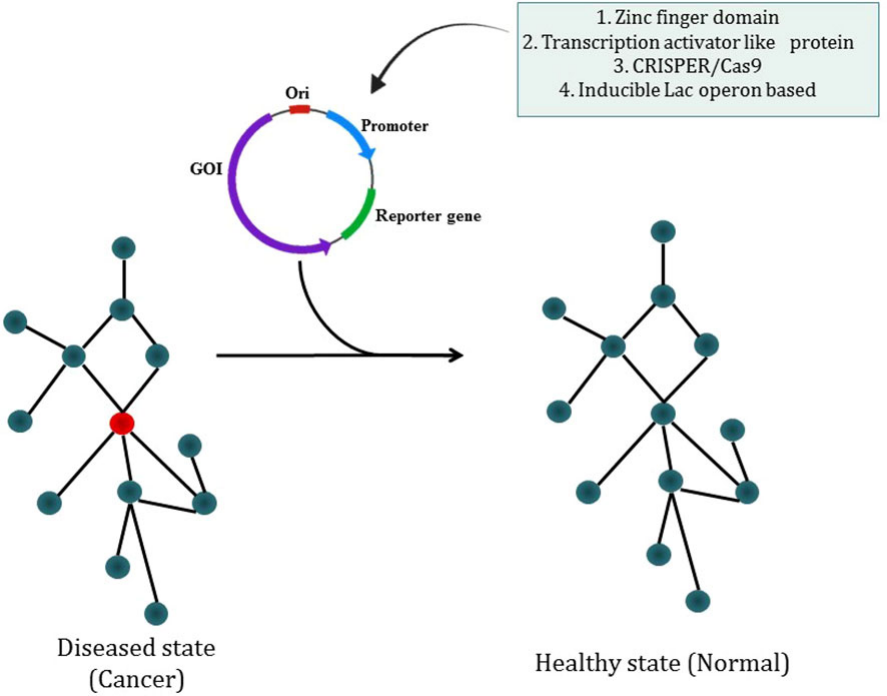
Synthetic biology modulating diseased state into a healthy state through different types of synthetic circuit.

The most prevalent kind of synthetic circuit is the one based on transcriptional control, which comprises a DNA-binding component that can recognize a promoter DNA sequence and an actuator component that allows positive or negative transcription regulation. Cys2-His2 zinc finger domains are especially suitable for the creation of synthetic transcription factors as they are often arranged as covalent tandem repeats, enabling the identification of long asymmetrical sequences (235). Researcher have described the apparent modular recognition of DNA by transcription activator-like (TAL) effectors, which is more amenable to DNA targeting and this proteins contain tandem polymorphic amino acid repeats independently which specify a single, contiguous nucleotide in the DNA target (236). The types of synthetic circuit are shown in **Figure 7**. The engineered zinc-finger protein transcription factors (ZFP TFs) are designed to fuse the human GM-CSF gene regulatory region to upregulate the expression of GM-CSF inserted in ONYX-411 for cancer treatment (237). CRISPR/Cas9 technology is used in gene circuits as they regulate gene expression. In CRISPR/Cas9,via Watson-Crick base pairing between the guide RNA and the target DNA, Cas9 is able to identify its target site and this Cas9 can target virtually any DNA sequence by changing the guide RNA’s 20 nucleotide targeting sequence (238) which makes CRISPR/Cas9 easier to use. But soon a deactivated Cas9 (dCas9) introduced with D10A, H841A two point mutations which nullify the protein’s endonuclease activity while retaining its potency to associate with gRNA and to be recruited on a particular DNA sequence. dCas9 can transcriptionally inhibit or activate target genes which are accordingly known as CRISPRi or CRISPRa (239). The study has reported the use of CRISPR-Cas9 based synthetic circuit for bladder cancer therapeutics. The engineered modular AND gate circuit consists of a cancer specific promoter of hTERT gene with hUPII bladder specific promoter to specifically recognize the bladder cancer cell. The hUPII promoter drives gRNA targeting lacI while the hTERT promoter drives dCas9.The output transgene was released from LacI-mediated repression as a result of the dCas9-gRNA complex’s suppression of lacI. Because of this, the output transgene was only observed in bladder cancer cells, which had active hTERT and hUPII (240). The dual-promoter integrator is one example of a gene circuit that targets cancer. In DPI circuit inputs are two cancer-specific promoters and use a transcriptional AND gate inspired by the mammalian two-hybrid system for computing output (241).

RNA plays crucial regulatory roles in a variety of biological systems, thus scientists are designing the RNA based regulatory circuit in cells. An example for RNA based synthetic circuits for translation control is miRNA based circuits. MiRNAs are likely fine-tuners rather than two-state on/off controllers of gene expression in nature. Thus, synthetic biology could benefit from it. Franscisco et al. reported construction of a synthetic circuit with two module miRNA system in *Chlamydomonas* which was a negative switch for silencing the genes. This constructed circuit can work under both constitutive promoter and inducible promoter (242). Another study has reported the synthetic circuit with miR-146a in leishmaniasis using lac operon based system. The on and off state depends on the presence of an inducer such as IPTG which leads to miR-146 expression (243). Liliana Wroblewska designed a circuit which identifies miRNAs expressions in HeLa cells. miRNA such as miR-21(High), miR-141(low), miR-142-3p and miR-146a are mentioned as specific for HeLa cell and this identification helps to differentiate within different cells. Further driving the pro-apoptotic gene (BAX) expression as circuit output could specifically target HeLa cells (Liliana wroblewska 2015). However, it was also found that the designed synthetic sensor can sense the expression profile of particular protein such as p53 in cancer cell. They have shown the decline of p53 correlates with the increase in HSV-TK expression (244). Genetic circuits with post translational control has also been reported in study (245). The synthetic biology can modulate treatment landscape as one advantage of synthetic biology is the speed with which one can design a circuit with therapeutic candidates for cancer. Further preclinical studies are needed to authenticate particular genetic circuits.

## 11 Conclusion

Many believe that immunotherapy has great promise in the battle against a variety of malignancies. The immune system may be trained to spot cancer if the proper stimuli are applied. Checkpoint inhibitors, which effectively act as stop signs that prevent T cells from destroying cancer cells, are some of the greatest instances of immunotherapy. Antibodies have been created recently that essentially block such inhibitory signals, enabling the immune system to combat cancer. Despite the efficacy and potency of the available immunotherapies, the dearth of the tumor specific antigens or the substances which can trigger a specific immune response to a kind of cancer remains as a great hurdle. On a concluding note, instead of trying to treat the entire body systemically, considerably more specialized, targeted immunotherapies that operate locally at the tumour site are required. Additionally, a variety of immunotherapies can be included in an individual package, allowing for the stimulation of an array of immune responses. By customizing synthetic circuits that are capable to trigger the desired immune response more accurately as compared to the available immunotherapy patterns might prove as a better therapeutic regimen.

## Author contributions

SS, NS and PG conceptualised the idea. NS and PG participated in writing of the manuscript along with figure preparation. SS, NS and PG edited the manuscript. All authors contributed to the article and approved the submitted version.

## Glossary

**Table:**

TGFa	Transforming growth factor a
VEGF	Vascular endothelial growth factor
BER	Base excision repair
NER	Nucleotide excision repair
HR	Homologous recombination
NHEJ	Non homologous end joining
MMR	Mismatch repair
EMT	Epithelial- mesenchymal transition
TGFb	Transforming growth factor b
PDGF	Platelet derived growth factor
EGF	Epidermal growth factor
CTGF	Connective tissue growth factor
FGF	Fibroblast growth factor
NOX4	NADPH oxidase 4
PD1	Programmed cell death protein 1
PDL1	Programmed death ligand 1
CTLA4	Cytotoxic T-lymphocyte associated antigen 4
EGFR	Epidermal growth factor receptor
ROS1	Reactive oxygen species 1
KRAS	Kristan rat sarcoma virus
ATR	Ataxia-telangiectasia-mutated-and-Rad3-related kinase
CRISPR	Clustered regularly interspaced short palindromic repeat
CAR-T	Chimeric antigen receptor T-cell therapy
NSCLC	Non small cell lung cancer
HER2	Human epidermal growth factor receptor 2
MET	Mesenchymal- epithelial transition factor
BRAF	v-raf murine sarcoma viral oncogene homolog B1
NRTK	Neurotrophic tyrosine receptor kinase
ALK	Anaplastic lymphoma kinase
HMGB1	High mobility group protein B1
ULK1/2	Unc-51 like autophagy activating kinase
mTORC1	Mammalian target of rapamycin complex 1
PHD1/2	Pyrol hydroxylases ½
HIF1a	Hypoxia Inducible Factor 1 Subunit Alpha
ZEB	Zinc finger E-box binding homeobox 1
PTEN	Phosphatase and tensin homolog
RUNX1	runt-related transcription factor 1
YAP	yes-associated protein 1
TNFa	Tumor necrosis factor alpha
IFNg	Interferon gamma
DGCR8	DiGeorge syndrome critical region 8
IL6	Interleukin 6
TNFAIP1	Tumor necrosis factor alpha induced protein 1
SMAD4	Mothers against decapentaplegic homolog 4
FOXO	Forkhead box O
NFkB	Nuclear factorkB
MCM	Minichromosome maintenance proteins
CCND3	Cyclin D3
CCD20	Central core disease
GATA6	GATA binding protein 6
NOVA2	Neuro oncological ventral antigen 2
CXCL1/2	C-X-C Motif Chemokine Ligand 1
AXL	AXL receptor tyrosine kinase
B7-H3	B7 Homolog 3
